# Volumetric MR-guided high-intensity focused ultrasound ablation to treat uterine fibroids through the abdominal scars using scar patch: a case report

**DOI:** 10.1186/s40349-016-0064-9

**Published:** 2016-08-11

**Authors:** Ying Zhu, Bilgin Keserci, Antti Viitala, Juan Wei, Xuedong Yang, Xiaoying Wang

**Affiliations:** 1 Radiology Department, Peking University First Hospital, 8 Xishiku Street, Beijing, 100034 China; 2 Philips Healthcare, Seoul, South Korea; 3 Philips Healthcare, Vantaa, Finland; 4 Philips Research China, Shanghai, China

**Keywords:** Scar patch, Uterine fibroid, Volumetric MR-guided high-intensity focused ultrasound

## Abstract

**Background:**

Abdominal scars pose a challenge in magnetic resonance-guided high-intensity focused ultrasound (MR-HIFU) therapies, limiting patient selection and increasing the risk of skin burns. Especially, scars arising from longitudinal incisions are problematic as they usually lie medially at the lower abdomen where the ultrasound beam has to go through. Volumetric sonication has been shown to efficiently enlarge the ablated volume per sonication, but they nevertheless require more thermal energy to be deposited per sonication which increases the temperature in the near-field area located between the transducer and the target region.

**Case presentation:**

The scar patch was used in three patients undergoing MR-HIFU ablation of fibroids using volumetric technique, one with transverse incision and the other two with longitudinal incision. No severe adverse effects were observed. The relative shrinkage of the fibroid of these patients at 6-month follow-up were 67, 78, and 59 %, respectively.

**Conclusions:**

Our preliminary experience suggests that the use of scar patch on MR-HIFU ablation of fibroids using volumetric technique provides an effective treatment option for patients who were previously excluded from MR-HIFU treatment due to the abdominal scars.

## Background

Magnetic resonance-guided high-intensity focused ultrasound (MR-HIFU) which allows for 3D treatment planning and feedback of temperature deposition in the area to be treated is an emerging therapy technique which uses focused ultrasound to heat and coagulate tissue deep within the body, with minimal damage to surrounding tissues. A number of studies have shown the clinical effectiveness of MR-HIFU for uterine fibroid treatment [1–8]. The conventional approach is performed by iterative sonication of a single focal point with each sonication followed by a cooling period. However, with this approach, a relatively large portion of the delivered energy is lost via diffusion of heat out of the small targeted region, and long treatment duration is required. Recently, a new volumetric ablation technique [9–11] has been introduced. This technique is able to utilize the outwards-diffused heat energy, while inducing relatively large ablation zones with an axial diameter of 4–16 mm since the focus of the ultrasound beam is electronically steered along a trajectory comprising of multiple outward-moving concentric circles. However, the energy deposition per sonication in this technique is higher than in the conventional one. Since all biological tissues absorb ultrasound energy to various extents, the increase in deposited energy might inevitably lead to higher temperature rise in the near field of the ultrasound beam path [12, 13].

The presence of abdominal scars may limit the access to the target area and increase the risk of near field damage during the MR-HIFU therapy. Until recently, the presence of scars in the ultrasound beam path was considered to be a contraindication for MR-HIFU in patients with uterine fibroids, as their presence can induce local skin heating leading to skin burns [3, 14–16]. At least one case with full-thickness skin burn at the location of previous laparoscopic scar necessitating a referral for plastic surgery has been reported [17, 18].

Zaher et al. [17] described a mitigation technique which helps to visualize the location of the scar and therefore facilitates therapy planning where the beam path does not go through the scar. This however is not practical with patients having longitudinal incision, as it is very difficult to avoid sonications through the area if the scar is located medially at the abdomen. Gorny et al. [19] proposed the use of acoustic patches on the skin to reflect the ultrasound energy from the scar. Yoon et al. [20] confirmed that the use of these scar patches can be a viable solution. In their study among 20 patients, about 57 % of the sonications in each treatment were through the scar patch, and only in one of the treatments (5 %), it was possible to tilt the beam to totally bypass the scar. Although two patients had minor red spots on the skin surface after the treatment and three other patients had slight hyperemic changes in the abdominal muscle, no serious adverse events were reported.

The purpose of this study is to assess the safety and technical feasibility of scar patch usage on MR-HIFU ablation of fibroids using volumetric technique. To the best of the authors’ knowledge, this is the first publication to report the use of the scar patches during MR-HIFU therapy on uterine fibroids using volumetric technique.

## Case presentation

### Materials and methods

As a part China Clinical Trial for Therapeutic MR-HIFU Ablation of Uterine Fibroids (sponsored by Philips Healthcare, clinicaltrials.gov identifier NCT01588899), a total of 58 MR-HIFU therapies were conducted at our institute. In three patients, the scar patch, in accordance with manufacturer’s instructions, labeling, and trial protocol, was used to prevent local skin heating which might lead to skin burns. All therapies were conducted using extracorporeal MRI-HIFU system (Sonalleve V2 MR-HIFU system) in combination with 3.0 T MR scanner (Achieva, Philips Healthcare, Amsterdam, The Netherlands). Three patients in our case report, one with transverse incision, the other two with longitudinal incision, received the same imaging protocols and treatment procedures. As shown in Table 1, the imaging protocols for screening, HIFU treatment, and 6-month follow-up after HIFU therapy are mainly including 3D sagittal T2-weighted turbo spin echo (T2W-TSE, fast field echo (FFE) and a contrast-enhanced T1-weighted turbo spin echo (T1W-TSE). The MR sequence used for temperature mapping is an RF-spoiled segmented echo planar imaging sequence: EPI factor = 11, repetition time TR = 37 ms, echo time TE = 19.5 ms, 121-binomial water-selective excitation.

**Table 1:** Imaging protocols

Examination	Sequence	Aim	Imaging plane	TR (ms)	TE (ms)	FA (°)	Section thickness (mm)	Field of view (mm)	Matrix size	TA (s)
Screening	3D T2 TSE	Uterine fibroid	Sagittal	1500	165	90	3	250 × 250	176 × 173	177
	FFE	Scar visualization	Coronal	3.4	1.74	10	2	200 × 200	172 × 173	79
	CE-T1 TSE*	Uterine fibroid	Coronal	5.5	2.7	12	2.5	250 × 250	192 × 192	130
Treatment	3D T2 TSE	Treatment planning	Sagittal	1550	150	90	1.6	250 × 250	176 × 173	124.9
	FFE	Scar and scar patch	Coronal	3.4	1.74	10	2	200 × 200	172 × 173	79
	EPI	Temperature mapping	Coronal, sagittal	37	19.5	19	7	400 × 400	160 × 160	2.9
	CE-T1 TSE*	Non-perfused volume	Coronal	5.5	2.7	12	2.5	250 × 250	192 × 192	130
6-month follow-up	3D T2 TSE	Uterine fibroid	Sagittal	1487	165	90	2.5	250 × 250	176 × 173	140
	CE-T1 TSE*	Non-perfused volume	Coronal	5.5	2.7	12	2.5	250 × 250	192 × 192	130

Depilation of the lower abdomen was conducted 1 day before the therapy, and Foley catheter was inserted just before the treatment. On the treatment day, patients were placed in a prone position before acquiring the pretreatment MR images for HIFU therapy planning. Then, the treatment cells were placed on the T2W planning images by carefully considering safety margins from the borders of the treatment cells to capsule of the fibroid and the critical organs such as sacral bone which were 1.5 and 4 cm, respectively. When necessary, urinary bladder filling with normal saline solution and/or rectal filling with US gel was performed to move the path of the sonication beam out of the scar area or to displace small bowel loops.

The binary feedback technique [9] was applied to perform volumetric ablation. The ablation zone (i.e., treatment cell) is ellipsoidal in shape and can be chosen as 4, 8, 12, 14, or 16 mm in axial dimension and 10, 20, 30, 35, or 40 mm in longitudinal dimension, respectively. Sonication power level (140–300 W) was determined based on the results of an initial test sonication with low power (60 W) and adjusted in an iterative manner based on the results of the previous sonications. Patients were asked to press a hand-held “stop button” if they experienced intolerable pain, and their movements and organ motions were monitored during the treatment by placing multiple fiducial markers at the boundary of the uterus and the pelvic bones on 3D T2W planning data. HIFU ablation was monitored during the sonications with multi-plane MR thermometry utilizing the proton resonance shift technique [21]. Three coronal planes and one sagittal plane through the target area together with one coronal plane positioned over the abdominal muscle were utilized.

The scar patches (QuickCover US Protective Cover) used in this study were made out of polyethylene foam with the size of 8 mm × 120 mm. The back of the sheet is self-adhesive to ensure that the patch does not move during the therapy. Once attached on the skin, the patch creates an ultrasound-reflecting air layer, thus preventing the ultrasound energy from reaching the scar tissue immediately behind the patch. Patches were clearly visible on the MR images due to the air contained within the material.

## Results

### Case 1

**Fig. 1:**
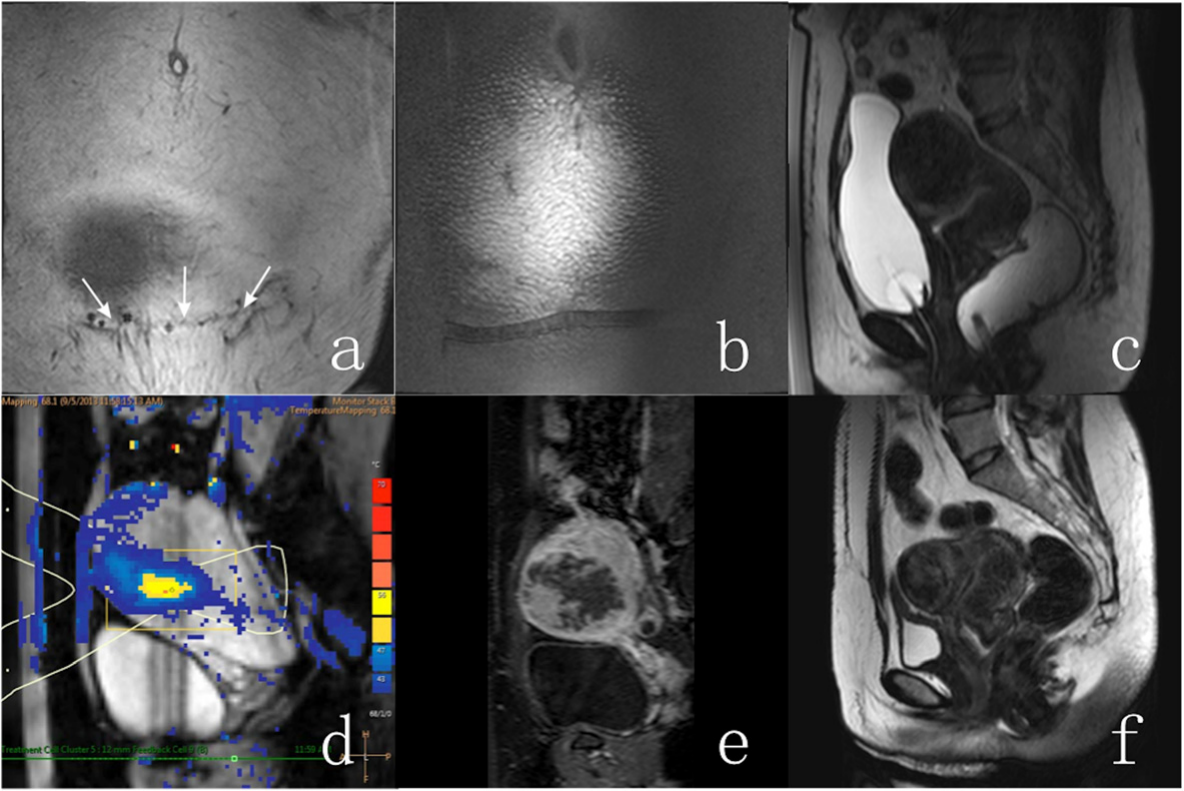
A 41-year-old woman who had a transverse incision scar presented with symptoms of menorrhagia, pelvic pressure, and urinary frequency was treated with MRI-guided high-intensity focused ultrasound ablation using scar patch. **a** Scar scan showing the orientation of the scar within the abdominal fat layer (scar location identified with white arrows). **b** Scar scan showing the air-containing scar patch at the patient’s skin. **c** T2w planning image acquired prior to the therapy. **d** PRF Thermometry scan acquired during one of the sonications. **e** T1w gadolinium-enhanced image acquired immediately after the therapy showing the induced necrosis. **f** T2w planning image at 6-month follow-up

A 41-year-old woman presented with symptoms of menorrhagia, pelvic pressure, and urinary frequency, with uterine fibroid symptom severity score (SSS) of 24. MRI study revealed two fibroids, one of which was chosen as a clinical target based on the symptom profile. Patient had a transverse incision scar on her lower abdomen (Fig 1a, b). Targeted fibroid was intramural, located at the anterior wall, and had a volume of 120 ml (Fig 1c). Fibroid was homogenous and iso-intense to muscle on the T1W images, hypo-intense to muscle on the T2W images, and demonstrated moderate and homogeneous enhancement on gadolinium-enhanced T1W image.

The treatment time from first to last sonication was 165 min. Good temperature rise was seen clearly at the focal area on sagittal temperature slice (Fig 1d), indicating that presence of the scar patch within the beam path did not affect the focus quality negatively. No obvious heating could be seen at the scar location. Delayed contrast-enhanced T1W images collected immediately after ablation showed that the immediate non-perfused volume (NPV) was 44 % (Fig 1e). Following the treatment, slight reddening of the skin at the upper abdomen, away from the scar patch area was observed. However, no areas of abnormal enhancement within the subcutaneous tissue or the regions of the scar were identified. The volume and shrinkage of the fibroid at 6 months follow-up were 39 ml and 67 %, respectively (Fig 1f). The patient reported that urinary frequency had completely resolved and the patients’ SSS reduced to 12.

### Case 2

**Fig. 2:**
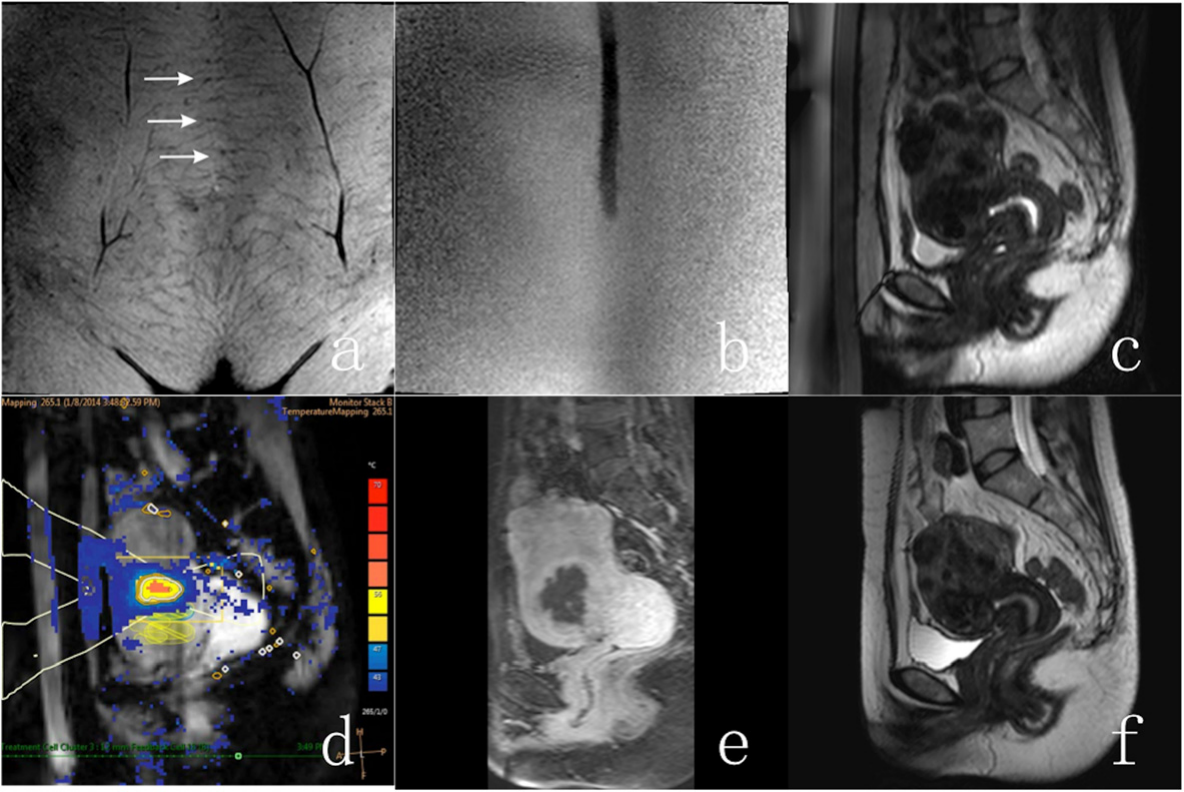
A 49-year-old woman who had a longitudinal incision presented with symptoms of menorrhagia, pelvic pressure, and urinary frequency was treated with MRI-guided high-intensity focused ultrasound ablation using scar patch. **a** Scar scan showing the orientation of the scar within the abdominal fat layer (scar location identified with white arrows). **b** Scar scan showing the air-containing scar patch at the patient’s skin. **c** T2w planning image acquired prior to the therapy. **d** PRF Thermometry scan acquired during one of the sonications. **e** T1w gadolinium-enhanced image acquired immediately after the therapy showing the induced necrosis. **f** T2w planning image at 6-month follow-up

A 49-year-old woman presented with symptoms of menorrhagia, pelvic pressure, and urinary frequency, with SSS of 22. MRI study revealed multiple fibroids, one of which was chosen as a clinical target based on the symptom profile. Patient had a longitudinal incision scar on her lower abdomen (Fig 2a, b). Targeted fibroid was located in the anterior wall and had a volume of 65 ml (Fig 2c). Fibroid was mostly homogenous and iso-intense to muscle on the T1W images and hypo-intense to muscle on the T2W images. T2W images showed also hyper-intense fascia within the fibroid.

The treatment time from first to last sonication was 112 min. Good temperature rise was seen clearly at the focal area on sagittal temperature slice (Fig 2d), and no obvious heating could be seen at the scar location. No skin reddening or other near field related adverse effects were observed. The NPV immediately after treatment was 41 % (Fig 2e). The volume and shrinkage of the targeted fibroid at 6-month follow-up were 14 ml and 78 % (Fig 2f). The SSS reduced to 13 at 6-month follow-up.

### Case 3

**Fig. 3:**
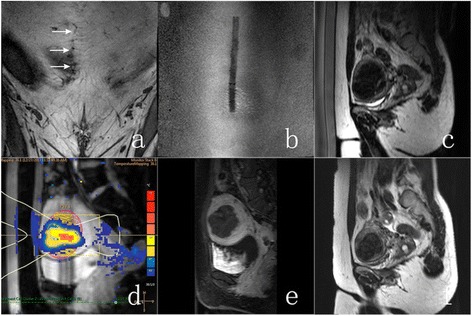
A 43-year-old woman who had a longitudinal incision presented with menorrhagia was treated with MRI-guided high-intensity focused ultrasound ablation using scar patch. **a** Scar scan showing the orientation of the scar within the abdominal fat layer (scar location identified with *white arrows*). **b** Scar scan showing the air-containing scar patch at the patient’s skin. **c** T2w planning image acquired prior to the therapy. **d** PRF thermometry scan acquired during one of the sonications. **e** T1w gadolinium-enhanced image acquired immediately after the therapy showing the induced necrosis. **f** T2w planning image at 6-month follow-up

A 43-year-old woman presented with menorrhagia, with SSS of 14. MRI study revealed longitudinal incision on her lower abdomen (Fig 3a, b) and multiple fibroids, one of which was chosen as a clinical target based on the symptom profile. Targeted fibroid was located in the anterior wall and had a volume of 71 ml (Fig 3c). The fibroid was mostly homogenous and iso-intense to muscle on the T1W images and hypo-intense to muscle on the T2W images with hyper-intense linear separation on T2W images.

The treatment time from first to last sonication was 101 min. Good temperature rise was seen clearly at the focal area on sagittal temperature slice (Fig 3d), and no obvious heating could be seen at the scar location. The NPV immediately after treatment was 80 % (Fig 3e). The volume and shrinkage of the fibroid at 6-month follow-up were 29 ml and 59 %, respectively (Fig 3f). The SSS at 6-month follow-up was 12.

## Discussion

In MR-HIFU treatments, the radiologists and/or gynecologists are trying to avoid passing US energy through the scar by tilting the beam around it to reduce risk of near-field damage. Abdominal scars pose a challenge in MR-HIFU therapies, possibly limiting patient selection and increasing the risk of skin burns. Yoon et al. [20] reported the use of scar patches in MR-HIFU treatment of uterine fibroids (19 patients with transverse incision and 1 patient with longitudinal incision) with point-by-point technique by showing low incidence of minor adverse events without severe adverse events comparing with previous studies without a scar patch [3, 5, 22, 23].

In our report with volumetric technique, there were three patients, one with transverse incision, the other two with longitudinal incision, all of whom received the same imaging protocols and treatment procedures. In sonications where beam path went through a scar, temperature images were followed carefully to detect any unwanted heating in the scar region. Sufficient cooling times were observed between sonications to reduce the possibility of damage due to accumulated heating. Good communication with the patient was ensured to obtain immediate information of any abnormal sensations. Volumetric MR-HIFU ablation therapy was technically successful in all three cases. Focus quality was not visibly affected by the presence of the scar patch, which indicates that the scar patch did not significantly reduce the efficacy of energy delivery to target, even though it blocks a small portion of the beam path. NPVs obtained in these three therapies were 44, 41, and 80 %, while 6-month fibroid shrinkages were 67, 78, and 59 %, respectively, indicating that clinical effectiveness is not hampered by the presence of the scar patch either. Reduced symptom severity score for each patient at 6-month follow-up also demonstrated the relief of subjective symptoms. In addition, there was no thermal injury of the abdominal muscle or subcutaneous fat layer.

Our study has some limitations. First, given all the fibroids enrolled were Funaki type 1 with hypo-vascularity [24], further research should be conducted to demonstrate the feasibility of using scar patches when the volumetric method is applied to fibroids of types 2 and 3, which are more vascular and typically require higher sonication energies. Second, we currently report results of only three cases. A prospective study with larger patient population is still needed.

## Conclusion

In conclusion, our preliminary experience suggests that the use of scar patch on MR-HIFU ablation of fibroids using volumetric technique provides an effective treatment option for patients who were previously excluded from MR-HIFU treatment due to the abdominal scars.

## Abbreviations

FFE, fast field echo; MR-HIFU, magnetic resonance-guided high-intensity focused ultrasound; NPV, non-perfused volume; SSS, symptom severity score; T1W-TSE, T1-weighted turbo spin echo; T2W-TSE, T2-weighted turbo spin echo
